# Detection of enteric pathogens in young children before and during acute gastroenteritis: results from a prospective German birth cohort study (LoewenKIDS)

**DOI:** 10.1007/s15010-025-02670-1

**Published:** 2025-10-20

**Authors:** Chiara Lincetto, Felipe Romero-Saavedra, Diana Laverde, Riccardo Lincetto, Melanie Meyer-Buehn, Bianca Klee, Cornelia Gottschick, Rafael Mikolajczyk, Johannes Huebner, Tilmann Schober

**Affiliations:** 1 Division of Pediatric Infectious Diseases, Dr. von Hauner Children’s Hospital, Ludwig Maximilians University, Munich, Germany; 2 Independent Researcher, Padova, Italy; 3 Institute for Medical Epidemiology, Biometrics and Informatics (IMEBI), Interdisciplinary Centre for Health Sciences, Medical Faculty of the Martin Luther University Halle-Wittenberg, Halle (Saale), Germany

**Keywords:** Acute gastroenteritis in children, Gastrointestinal pathogens, Enteric viruses, Diarrhea

## Abstract

**Purpose:**

To identify enteric pathogens in pediatric acute gastroenteritis (AGE) and assess their etiological relevance by comparison with samples during asymptomatic period.

**Methods:**

Children < 2 years of age (*n* = 89) were prospectively enrolled as part of the population-based birth cohort LoewenKIDS. Asymptomatic stool samples were collected regularly, and symptomatic samples were collected after the occurrence of > 3 loose stools and/or one vomiting in 24 h. Intraindividual pairs of symptomatic and preceding asymptomatic samples for each child were analyzed for 25 enteric pathogens via multiplex real-time RT-PCR.

**Results:**

Enteric viruses were detected in 64% (57/89) of symptomatic samples and significantly associated with gastrointestinal symptoms (Odds Ratio [OR] 3.9; 95% Confidence Interval [CI] 2.1–7.3). The most common viruses in AGE were norovirus (Genogroups GI and GII) (36%, 32/89) and adenovirus (27%, 24/89). Bacteria were detected in 46% (41/89) of symptomatic samples and 43% (38/89) of asymptomatic ones, with no association to symptoms (OR 1.1; 95% CI 0.6-2). The most common bacteria in AGE were Enteropathogenic *Escherichia coli* (28%, 25/89) and *Clostridium difficile* (16%, 14/89). *Dientamoeba fragilis* was the only detected parasite in AGE (7%, 6/89), and was not associated with symptoms (OR 1.4; 95% CI 0.4–5.5). Pathogen loads in symptomatic and asymptomatic pairs correlated with symptoms for norovirus GII, astrovirus and sapovirus (each *p* < 0.01), but not for other pathogens.

**Conclusion:**

This study supports the clinical significance of detection of viral pathogens in young children with acute gastroenteritis and without relevant comorbidities in high-income countries, but limits the significance of enteric bacterial and parasitic pathogens detection, partly due to constraints in their specific identification.

**Trial registration:**

Clinicaltrials.Gov Identifier: NCT02654210.

**Supplementary Information:**

The online version contains supplementary material available at 10.1007/s15010-025-02670-1.

## Introduction

Acute gastroenteritis (AGE) is a common, often self-limited condition, typically caused by infection by viruses, bacteria, or parasites. Characterized by inflammation of the stomach and intestines, AGE presents with symptoms such as diarrhea, abdominal pain, nausea, vomiting, fever, and dehydration [1].

AGE poses a significant burden on global health, particularly affecting children under 5 years of age, among whom diarrhea ranks as the third leading cause of death worldwide [2]. This is evident in low-income countries due to limited access to clean water, sanitation, and healthcare services [3, 4]. Even in high-income countries, AGE remains a leading cause of morbidity, resulting in frequent hospitalizations, outpatient visits, and substantial healthcare costs [5–7].

A diverse array of pathogens can contribute to AGE, with enteric viruses, notably rotavirus, norovirus, and adenovirus, accounting for a majority of cases worldwide [8, 9]. While bacterial causes are less common, they can cause severe gastroenteritis outbreaks [10, 11], whereas parasites are considered to primarily pose challenges in low- and middle-income countries [12, 13]. Notably, public health interventions, such as rotavirus vaccination, have altered the etiology of AGE, with norovirus emerging as a predominant cause following the decline in rotavirus cases [8, 14]. In parallel, efforts to explain the estimated 30–40% of AGE cases that remain without a known etiology have increasingly focused on *Picornaviridae* viruses. Among these, enteroviruses are gaining recognition as potential contributors to pediatric AGE across diverse regions [15–17].

Accurate identification of the pathogen responsible for AGE is important for effective patient management, especially given the symptoms do not reliably identify the causative agent [9]. While recent diagnostic advancements have enhanced our understanding of pathogen prevalence and behavior, attributing disease solely to a specific organism can be misleading - particularly in the presence of multiple pathogens [18, 19]. Moreover, asymptomatic infections and transmission from carriers without symptoms have further complicated our understanding of the causal relationships between specific pathogens and disease. An example of this is infection with enteroaggregative *Escherichia coli* (EAEC), commonly associated with AGE, has not shown a consistent relationship with disease in longitudinal and case-control studies both in high- and lower-middle-income countries [20, 21]. While identifying the precise etiology may not always alter clinical management, in some circumstances it can help prevent unnecessary antibiotic use, thus averting the emergence of bacterial resistance and disruption of the microbiome [1].

In this study, we aimed to elucidate the role of gastrointestinal pathogens in the development of AGE. We analyzed enteric infections in children, comparing samples collected during AGE episodes with samples collected before symptom onset. This comprehensive approach sets our study apart, as previous investigations have often focused solely on symptomatic samples [18, 22, 23] or on specific pathogenic agents or agent types [21, 24–27]. To our knowledge, alongside a study from the Netherlands that, unlike this work, focused specifically on day care attendees [28], this is the first longitudinal study to comprehensively examine the role of a broad panel of pathogens in a pediatric cohort with AGE in a high-income setting.

## Materials and methods

### Study cohort

The LoewenKIDS study (Clinicaltrials.Gov Identifier: NCT02654210) is a population-based observational birth cohort study. It prospectively recruited 782 infants between November 2014 and February 2018 in five study locations in Germany. Participants were enrolled during the antenatal period and up to three months of age, and are being followed up until 15 years of age. The study design, methods of recruiting, and data collection are all described in full elsewhere [29]. If diarrhea, defined as the occurrence of at least four loose stools within 24 h, and/or vomiting appeared at least once within 24 h, a stool sample was taken for gastrointestinal symptoms. Routine, asymptomatic stool samples were collected once a year up to the age of six for the main cohort. For an intensified subcohort, routine stool samples were collected four times a year in the first two years and yearly afterwards. Parents filled in a daily symptom diary for gastrointestinal and respiratory symptoms.

In this study, we planned to analyze stool samples from 100 children. First, we selected children with high completeness of biosamples, questionnaires, and symptom diaries (at least 75%). Second, we selected children who had a symptomatic stool sample and had a corresponding entry in the symptom diary within the first two years of life. Criteria for the symptom diary entries included vomiting, diarrhea or a medical diagnosis of a gastrointestinal infection. Third, we checked whether an asymptomatic stool sample was available less than 120 days before the symptomatic sample, resulting in a final sample of 91 children. For each child, we analyzed both samples.

### Sample processing

Stool samples were stored at − 80 °C in RNASepar Solution (Biosepar). Upon thawing, around 150 mg of stool samples were homogenized in 1 ml ASL buffer (Qiagen) by vortexing for one minute and incubated for 10 min at room temperature (RT). Samples were then centrifuged for two minutes at 14,000 rpm in a benchtop centrifuge, and 800 µl of the supernatant was transferred in a new microcentrifuge tube. Nucleic acids were then extracted in a Nimbus automated system. Pathogen detection was performed via multiplex real-time RT-PCR, using the Allplex™ Gastrointestinal Panel Assays (Seegene). Cycle threshold (Ct) was determined for each pathogen. The panel includes 25 enteric pathogens, and specifically six viruses: adenovirus (subgenus F), astrovirus, norovirus GI, norovirus GII, rotavirus, sapovirus; 13 bacteria: *Aeromonas* spp., *Campylobacter* spp., *Clostridium difficile* (toxin B), *Salmonella* spp., *Shigella* spp./enteroinvasive *Escherichia coli* (EIEC *E. coli*), *Vibrio* spp., *Yersinia enterocolitica*, enteroaggregative *E. coli* (EAEC) (*aggR* locus), enteropathogenic *E. coli* (EPEC) (*eaeA* locus), *E. coli O157*, enterotoxigenic *E. coli* (ETEC) (*lt/st* locus), hypervirulent *C. difficile*, Shiga toxin-producing *E. coli* (STEC) (*stx1/2* locus); six parasites: *Blastocystis hominis*,* Cryptosporidium* spp., *Cyclospora cayetanensis*,* Dientamoeba fragilis*,* Entamoeba histolytica*,* Giardia lamblia.*

### Statistical analysis

Data analysis was performed using statistical packages in Python 3. Continuous variables are summarized as means ± standard deviations (SD). Categorical data are presented as counts and percentages. Odds ratios (ORs) with 95% confidence intervals (CIs) were calculated to assess associations. Clustering analysis was performed using k-means on the principal components obtained through Principal Component Analysis (PCA) of the full detection profile of samples, with the inverse of Ct values serving as a measure of pathogen load. Paired comparisons between asymptomatic and symptomatic samples were performed using mixed-effects models on the standardized inverse of Ct values (representing pathogen load), with sample type (asymptomatic vs. symptomatic) as a fixed effect and the individuals as a random intercept to account for the paired nature of the data (paired samples within individuals). A two-sided *p*-value < 0.05 was considered statistically significant.

### Ethical approval

All parents of study participants gave their written consent after receiving full information. The research protocol was accepted by the respective ethics committees of the Medical Faculty of the Martin Luther University Halle-Wittenberg (No. 2016-04), the Medical School Hannover (No. 6794) and the Ludwig Maximilian University Munich (No. 445 − 15), Germany.

## Results

### Description of the cohort

**Fig. 1:**
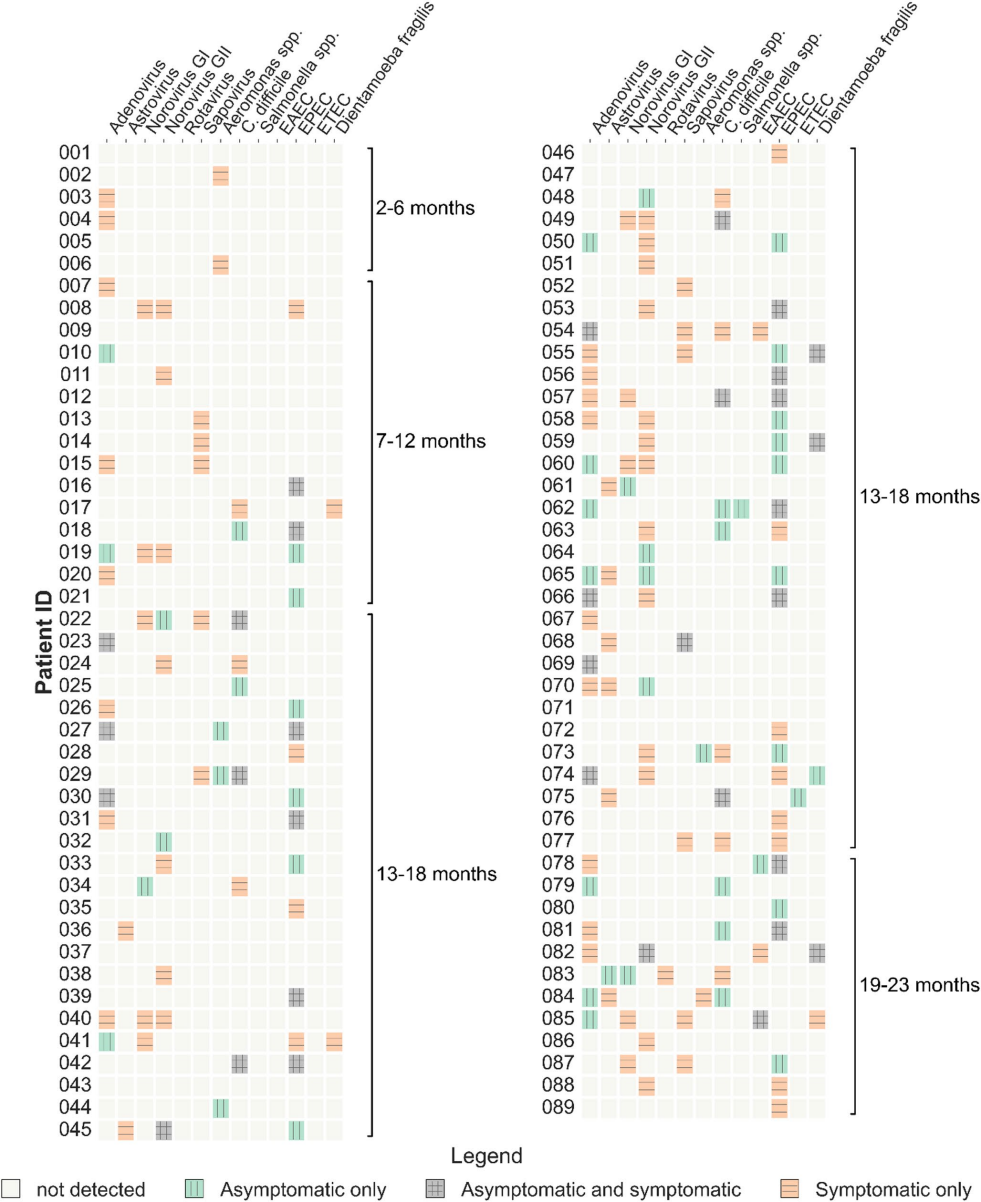
Pathogens detected in symptomatic and asymptomatic samples of individual children. Each row represents a child, ordered by increasing age at the time of symptomatic sample collection. For each child, a pathogen was detected either only in the symptomatic sample (orange with horizontal lines), only in the asymptomatic sample (green with vertical lines), or in both (grey with combined horizontal and vertical lines). Brackets represent different 6-month age intervals

This study included 91 children from the LoewenKIDS cohort who developed acute gastroenteritis (AGE) between 2015 and 2019. The mean age of children at AGE onset was 14 months (SD 4.4, range 2–23 months). For each of them, a stool sample collected at the time of AGE (symptomatic sample) and one obtained prior to AGE (asymptomatic sample) were analyzed for the presence of intestinal pathogens. Two symptomatic samples yielded invalid results for one of the panels. These and the corresponding asymptomatic samples were therefore excluded from all subsequent analysis. All results presented are based on data from 89 children. An overview of the full findings per child is presented in Fig. 1.

### Microbiological findings in AGE samples

AGE episodes were more frequently reported during the winter and autumn months (Supplementary Fig. 1a). We could detect at least one pathogen in 82% (73/89) of the symptomatic samples. Codetections were observed in 43% (38/89) of symptomatic samples, with up to four pathogens identified in individual cases. Overall, 28% (25/89) of the symptomatic samples were positive for two pathogens, 10% (9/89) for three pathogens, and 4% (4/89) for four pathogens (Table 1).

**Table 1:** Number of samples grouped by different enteric pathogens detection profiles and stratified by sample type, in a paired-sample cohort of 89 children with AGE. Data is presented as absolute counts and percentages among all children. The odds ratio (OR) quantifies the association between pathogen presence and symptomatic illness, indicating the likelihood of a sample being symptomatic rather than asymptomatic based on pathogen detection

Detection profile			Total *N*.	Symptomatic	Asymptomatic	OR (95% CI range)
**At least one pathogen**			**126**	73 (82.0%)	53 (59.6%)	3.1 (1.6–6.2)
	**1 pathogen**		**62**	35 (39.3%)	27 (30.3%)	1.5 (0.8–2.8)
	**Multiple pathogens**		**64**	38 (42.7%)	26 (29.2%)	1.8 (1.0–3.3)
		**2 pathogens**	**48**	25 (28.1%)	23 (25.8%)	1.1 (0.6–2.1)
		**3 pathogens**	**11**	9 (10.1%)	2 (2.2%)	4.9 (1.0–23.4)
		**4 pathogens**	**5**	4 (4.5%)	1 (1.1%)	4.1 (0.4–37.4)
**No pathogen detected**			**52**	16 (18.0%)	36 (40.4%)	0.3 (0.2–0.6)

The detection rates of viral, bacterial and parasitic pathogens in symptomatic samples were 64% (57/89), 46% (41/89), and 7% (6/89), respectively (Table 2). the most commonly detected pathogens were enteropathogenic *Escherichia coli* (EPEC) (28%, 25/89), adenovirus (27%, 24/89), followed by Norovirus GII (25%, 22/89), *Clostridium difficile* (16%, 14/89), and Sapovirus (13%, 12/89) (Table 2).

**Table 2:** Number of samples in which each Gastrointestinal pathogen was detected, in a paired-sample cohort of 89 children with AGE, stratified by sample type. Data is presented as absolute counts and percentages among all children of samples presenting with each pathogen. Odds ratio (OR) indicates association with symptoms, indicating the likelihood of a sample being symptomatic rather than asymptomatic based on pathogen detection

Pathogen	Total *N*.	Symptomatic	Asymptomatic	OR (95% CI range)
**Virus**	**85**	**57 (64.0%)**	**28 (31.5%)**	**3.9 (2.1–7.3)**
Adenovirus	41	24 (27.0%)	17 (19.1%)	1.6 (0.8–3.2)
Astrovirus	9	8 (9.0%)	1 (1.1%)	8.7 (1.1–71.1)
Norovirus GI	13	10 (11.2%)	3 (3.4%)	3.6 (1.0–13.6)
Norovirus GII	30	22 (24.7%)	8 (9.0%)	3.3 (1.4–7.9)
Rotavirus	1	1 (1.1%)	0 (0.0%)	-
Sapovirus	13	12 (13.5%)	1 (1.1%)	13.7 (1.7–107.8)
**Bacterium**	**79**	**41 (46.1%)**	**38 (42.7%)**	**1.1 (0.6–2.0)**
Aeromonas spp.	7	3 (3.4%)	4 (4.5%)	0.7 (0.2–3.2)
Clostridium difficile	27	14 (15.7%)	13 (14.6%)	1.1 (0.5–2.5)
Salmonella spp.	1	0 (0.0%)	1 (1.1%)	0.0 (0.0 - nan)
Enteroaggregative E. coli (EAEC)	5	3 (3.4%)	2 (2.2%)	1.5 (0.2–9.2)
Enteropathogenic E. coli (EPEC)	53	25 (28.1%)	28 (31.5%)	0.9 (0.5–1.7)
Enterotoxigenic E. coli (ETEC)	1	0 (0.0%)	1 (1.1%)	0.0 (0.0 - nan)
**Parasite**	**10**	**6 (6.7%)**	**4 (4.5%)**	**1.5 (0.4–5.5)**
Dientamoeba fragilis	10	6 (6.7%)	4 (4.5%)	1.5 (0.4–5.5)

### Microbiological findings in asymptomatic samples

Asymptomatic samples were collected at a median of eight weeks before AGE onset (interquartile range: 4.8–11.1 weeks). At least one pathogen was identified in 60% (53/89) of asymptomatic samples. Codetections were also common (29%, 26/89), with 26% (23/89) of asymptomatic samples showing two pathogens, 2% (2/89) three pathogens, and 1% (1/89) with four pathogens (Table 1).

In asymptomatic samples, the non-exclusive proportion of viral, bacterial, and parasitic detections was 32% (28/89), 43% (38/89), and 4% (4/89), respectively (Table 2). All detected pathogens, except rotavirus, were also found in at least one asymptomatic sample. The most common pathogens identified in asymptomatic samples were EPEC (31%, 28/89), adenovirus (19%, 17/89), *C. difficile* (15%, 13/89), and norovirus GII (9%, 8/89). (Table 2).

### Comparative analysis of symptomatic and asymptomatic samples

Pathogen profiles of symptomatic and asymptomatic samples were very similar and clustering analysis did not yield a clear separation between the two groups (Supplementary Fig. 2). Nevertheless, univariate analysis revealed differences between the groups, suggesting that odds of testing positive for at least one pathogen were higher in symptomatic samples compared to asymptomatic ones (OR 3.1, 95% CI 1.6–6.2). Based on univariate analysis, detection of multiple pathogens was also more common in symptomatic samples (OR 1.8, 95% CI 1.0–3.3) (Table 1).

When stratified by pathogen type, viral pathogens were more common in symptomatic compared to asymptomatic samples (OR 3.9, 95% CI 2.1–7.3), while parasites (OR 1.5, 95% CI 0.4–5.5) and bacteria (OR 1.1, 95% CI 0.6–2.0) were not (Table 2).

When examining intra-individual differences in pathogen profiles, no new pathogens were detected in 28% (25/89) of children experiencing AGE compared to their previous asymptomatic sample. In contrast, 72% (64/89) of sick children presented with new pathogens. More specifically, 44% (39/89) had a single new pathogen, while 28% (25/89) had multiple new pathogens (Table 3). The pathogen profile in symptomatic children was further complicated by the recurrent detection of pathogens in both sample types, observed in 31% (28/89) of cases (Fig. 1). Finally, 44% (39/89) of children had at least one pathogen in their asymptomatic sample that was no longer detectable at the time of AGE (Table 3). Notably, infants in the earliest months of life exhibited simpler pathogen profiles, with no observed codetections (Fig. 1).

**Table 3:** Number of cases where, within each child’s full detection profile, at least one pathogen was only detected in the symptomatic sample (“New detections”), exclusively detected in the asymptomatic sample (“Cleared detections”), or was detected in both samples (“Recurrent detections”), in a paired-sample cohort of 89 children that developed AGE. Data are presented as absolute counts and percentages of all children

Detection profile category		New detections	Recurrent detections	Cleared detections
**At least one pathogen**		64 (72%)	28 (32%)	39 (44%)
	**1 pathogen**	39 (44%)	23 (26%)	30 (34%)
	**2 pathogens**	19 (21%)	5 (6%)	7 (8%)
	**3 pathogens**	6 (7%)	0	2 (2%)
**No pathogen**		25 (28%)	61 (68%)	50 (56%)

### Viral detections

**Fig. 2:**
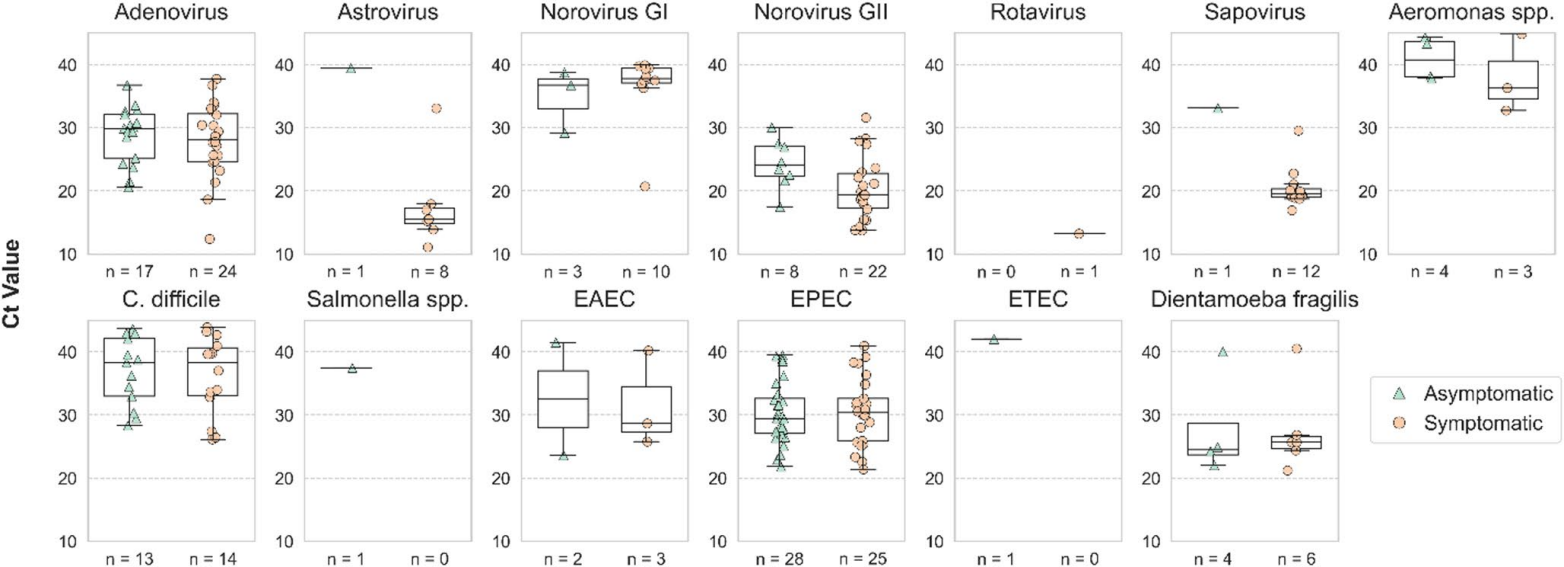
Pathogen loads (Ct values) in asymptomatic (green triangles) and symptomatic samples (orange circles). Boxplots show the quartiles of the dataset, the whiskers extend to points that lie within 1.5 interquartile ranges (IQRs) of the lower and upper quartile, and observations that fall outside this range are displayed as outliers. Comparisons between symptomatic and asymptomatic groups for each pathogen were conducted using the Mann-Whitney U test, accounting for the non-normal distribution of the data

All six viruses included in the panel were identified in the cohort. Viral pathogens were more frequently detected in symptomatic samples (Tables 2 and 4), and their viral loads were generally elevated compared to asymptomatic samples, though with some overlap (Fig. 2; Table 5). Codetections in virus-positive symptomatic samples were common (73%, 56/89) and occurred at comparable frequency in asymptomatic samples (60%, 18/89) (Table 6). Viral presence was distributed throughout the year, with higher trends in winter months and sporadic peaks in the summer (Supplementary Fig. 1b).

**Table 4:** Number of children carrying each pathogen in a paired-sample cohort of 89 children with AGE, further stratified by whether the pathogen was detected in their symptomatic samples, asymptomatic samples, or both. Data is presented as absolute counts and percentages among all children carrying that pathogen

Pathogen	Total *N*.	Symptomatic sample only	Both asymptomatic and symptomatic samples	Asymptomatic sample only
**Virus**				
Adenovirus	34	17 (50.0%)	7 (20.6%)	10 (29.4%)
Astrovirus	9	8 (88.9%)	0 (0.0%)	1 (11.1%)
Norovirus GI	13	10 (76.9%)	0 (0.0%)	3 (23.1%)
Norovirus GII	28	20 (71.4%)	2 (7.1%)	6 (21.4%)
Rotavirus	1	1 (100.0%)	0 (0.0%)	0 (0.0%)
Sapovirus	12	11 (91.7%)	1 (8.3%)	0 (0.0%)
**Bacterium**				
Aeromonas Spp.	7	3 (42.9%)	0 (0.0%)	4 (57.1%)
Clostridium difficile	21	8 (38.1%)	6 (28.6%)	7 (33.3%)
Salmonella Spp.	1	0 (0.0%)	0 (0.0%)	1 (100.0%)
Enteroaggregative E. Coli (EAEC)	4	2 (50.0%)	1 (25.0%)	1 (25.0%)
Enteropathogenic E. Coli (EPEC)	40	12 (30.0%)	13 (32.5%)	15 (37.5%)
Enterotoxigenic E. Coli (ETEC)	1	0 (0.0%)	0 (0.0%)	1 (100.0%)
**Parasite**				
Dientamoeba Fragilis	7	3 (42.9%)	3 (42.9%)	1 (14.3%)

**Table 5:** Comparison of pathogen loads (standardized inverse Ct values) between symptomatic and asymptomatic samples, for each individual. Paired analysis was performed by mixed-effects model, treating individuals as random effects. Data is presented as coefficients, representing the estimated differences in viral load between sample types (symptomatic vs. asymptomatic), and *p*-values, indicating the statistical significance of the estimated coefficients. Coefficient values range from 1, indicating the highest pathogen load in symptomatic samples, to -1, indicating the highest pathogen load in asymptomatic samples, and 0 when there is no difference in pathogen load between sample types. * *p* ≤ 0.05; ** *p* ≤ 0.01; *** *p* ≤ 0.001

Pathogen	Coefficient (95% CI Range)	*P*-Value	
**Virus**			
Adenovirus	0.239 (-0.0463–0.5243)	0.1007	
Astrovirus	0.396 (0.1064–0.6856)	0.0074 (**)	
Norovirus GI	0.2898 (-0.0026–0.5821)	0.0521	
Norovirus GII	0.4729 (0.1858–0.76)	0.0012 (**)	
Rotavirus	0.1503 (-0.1443–0.4449)	0.3172	
Sapovirus	0.5005 (0.2266–0.7744)	0.0003 (***)	
**Bacterium**			
Aeromonas Spp.	-0.0403 (-0.3357–0.2551)	0.7892	
Clostridium difficile	0.0427 (-0.1923–0.2778)	0.7217	
Salmonella Spp.	-0.1503 (-0.4449–0.1443)	0.3172	
Enteroaggregative E. Coli (EAEC)	0.0644 (-0.1304–0.2592)	0.5168	
Enteropathogenic E. Coli (EPEC)	-0.0763 (-0.3314–0.1788)	0.5579	
Enterotoxigenic E. Coli (ETEC)	-0.1503 (-0.4449–0.1443)	0.3172	
**Parasite**			
Dientamoeba Fragilis	0.0961 (-0.0686–0.2608)	0.2526	

**Table 6:** Number of detections of each pathogen in a paired-sample cohort of 89 children with AGE, stratified by sample type and codetection profile. Data is presented as absolute counts and percentages among the total number of detections of each pathogen per sample type

Pathogen	Symptomatic samples			Asymptomatic samples		
	Tot *N*.	Single pathogen	Multiple pathogens	Tot *N*.	Single Pathogen	Multiple pathogens
**Virus**	**77**	**21 (27.3%)**	**56 (72.7%)**	**30**	**12 (40.0%)**	**18 (60.0%)**
Adenovirus	24	9 (37.5%)	15 (62.5%)	17	5 (29.4%)	12 (70.6%)
Astrovirus	8	3 (37.5%)	5 (62.5%)	1	0 (0.0%)	1 (100.0%)
Norovirus GI	10	0 (0.0%)	10 (100.0%)	3	2 (66.7%)	1 (33.3%)
Norovirus GII	22	6 (27.3%)	16 (72.7%)	8	4 (50.0%)	4 (50.0%)
Rotavirus	1	0 (0.0%)	1 (100.0%)	0	0 (Nan%)	0 (Nan%)
Sapovirus	12	3 (25.0%)	9 (75.0%)	1	1 (100.0%)	0 (0.0%)
**Bacterium**	**45**	**14 (31.1%)**	**31 (68.9%)**	**49**	**15 (30.6%)**	**34 (69.4%)**
Aeromonas Spp.	3	2 (66.7%)	1 (33.3%)	4	1 (25.0%)	3 (75.0%)
Clostridium difficile	14	2 (14.3%)	12 (85.7%)	13	3 (23.1%)	10 (76.9%)
Salmonella Spp.	0	0 (Nan%)	0 (Nan%)	1	0 (0.0%)	1 (100.0%)
Enteroaggregative E. Coli (EAEC)	3	0 (0.0%)	3 (100.0%)	2	0 (0.0%)	2 (100.0%)
Enteropathogenic E. Coli (EPEC)	25	10 (40.0%)	15 (60.0%)	28	11 (39.3%)	17 (60.7%)
Enterotoxigenic E. Coli (ETEC)	0	0 (Nan%)	0 (Nan%)	1	0 (0.0%)	1 (100.0%)
**Parasite**	**6**	**0 (0.0%)**	**6 (100.0%)**	**4**	**0 (0.0%)**	**4 (100.0%)**
Dientamoeba Fragilis	6	0 (0.0%)	6 (100.0%)	4	0 (0.0%)	4 (100.0%)

When accounting for both of its genogroups (I and II) examined, norovirus was the most commonly detected virus in samples of children with AGE (36%, 32/89). Both genogroups were also detected in asymptomatic samples, although much less frequently (12%, 11/89). Univariate analysis confirmed that the presence of any of the two norovirus genogroups was clearly associated with symptoms (Table 2). However, viral loads in symptomatic samples were similar to those in asymptomatic ones (*p*-value: 0.0502 for norovirus GII) (Fig. 2). Paired analysis to compare pathogen loads between symptomatic and asymptomatic samples for each individual, performed using a mixed-effects model, confirmed an association between viral load and symptoms only for norovirus GII (*p*-value: 0.0012) (Table 5). Codetections in symptomatic samples were common as well (Table 6). Adenovirus was the second most common enteric virus found in AGE samples, yet it was often present in asymptomatic samples as well (27% and 19%, respectively), and at similar viral loads (Fig. 2). The association of Adenoviral detection with symptoms (OR 1.6, 95% CI 0.8–3.2) was the weakest among viral pathogens (Table 2). Sapovirus was the viral pathogen most clearly associated with symptoms (OR 13.7, 95% CI 1.7–107.7) (Table 2). It was detected only in symptomatic children (13%, 12/89) and in the asymptomatic sample of one of them, which was collected 7 weeks before AGE, and where viral load was much lower (Tables 1 and 4; Fig. 2). Similar findings were made for astrovirus (OR 8.7, 95% CI 1.1–71.1), which was detected in 9% (8/89) of children showing symptoms, and only in one asymptomatic sample, where the viral load was very low (Tables 1 and 4; Fig. 2). Paired analysis confirmed a significant positive association of viral load and symptoms for both sapovirus and astrovirus (*p*-values: 0.0003 and 0.007, respectively) (Table 5). Finally, rotavirus appeared only in one symptomatic sample.

### Bacterial and parasitic detections

Bacteria were more commonly detected in summer and spring (Supplementary Fig. 1b). None of the bacterial pathogens detected, namely (from the most abundant) enteropathogenic *E. coli* (EPEC), *C. difficile*, *Aeromonas* spp., enteroaggregative *E. coli* (EAEC), *Salmonella* spp. and enterotoxigenic *E. coli* (ETEC), had any association with symptoms (Table 2). Paired analysis performed to compare bacterial loads between symptomatic and asymptomatic samples for each individual confirmed the lack of a significant correlation between pathogen quantity and the clinical manifestation of AGE (Table 5). In contrast, most bacteria were detected at similar frequencies in both sample types (Tables 2 and 4). This was also observed for EPEC, which, overall, was the most abundant pathogen identified in our cohort (30%, 53/178). Average bacterial loads were similar across sample types (Fig. 2). Codetections were often observed both in symptomatic and asymptomatic samples (Table 6). Other bacteria included in the panel, specifically *Campylobacter* spp, *Shigella* spp./enteroinvasive *E. coli* (EIEC), *Vibrio* spp, *Yersinia enterocolitica*,* E. coli* O157, hypervirulent *C. difficile* and Shiga toxin-producing *E. coli* (STEC), were not detected in our cohort.

The only parasite detected was *Dientamoeba fragilis*, found in 7% (6/89) of symptomatic children. Of these, three also had the parasite in their pre-AGE asymptomatic samples, and it was additionally detected in the asymptomatic sample of one unrelated child (Tables 2 and 4). Similar to bacterial patterns, *D. fragilis* was more frequently observed during summer (Supplementary Fig. 1b). No significant correlation between parasite load and symptoms was found (Table 5). In each sample, codetections were also identified (Table 6). *Blastocystis hominis*, *Cryptosporidium* spp., *Cyclospora cayetanensis*, *Entamoeba histolytica* and *Giardia lamblia* were not detected in our cohort.

## Discussion

This longitudinal analysis is an integral component of the broader LoewenKIDS project, a prospective population-based observational birth cohort study conducted in five study locations across Germany. The primary aim of the LoewenKIDS project is to investigate the impact of bacterial colonization and infections in children and the association with the development of chronic diseases later in life [29]. This analysis focused on the detection of enteric pathogens in a sub-cohort of 89 children under two years of age who developed AGE during follow-up. Notably, the LoewenKIDS study design provided us with access to asymptomatic control samples collected from the same children prior to the onset of AGE symptoms. This access was critical in providing a better understanding of the pathogenicity of enteric microbes, setting our study apart from others lacking longitudinal pre-AGE information.

At least one pathogen was detected in 82% of samples obtained from children experiencing AGE, underscoring the suitability of the panel for identifying AGE-causative agents using self-sampling within a German cohort. Consistent with previous studies, viral pathogens were predominant, detected in 64% of acute gastroenteritis samples, followed by bacteria (46%) and parasites (7%) [24, 30]. Codetection rates were substantial at 43%, indicating a high prevalence of mixed infections, which might contribute to disease complexity. Norovirus (genogroups I and II together), enteropathogenic *Escherichia coli* (EPEC), and adenovirus, followed by *Clostridium difficile*, were the most frequently detected pathogens in symptomatic children. Similar pathogen distributions and codetection rates have been reported in prior studies from high-income countries, suggesting the consistency of these epidemiological patterns across diverse populations [18, 24, 30, 31].

Enteric viruses are a leading cause of gastroenteritis worldwide, and the analysis of our cohort confirm this observation. Rotavirus causes more severe gastroenteritis than most other enteric pathogens [8]. According to the 2016 Global Burden of Disease Study, rotavirus was by far the leading etiology responsible for diarrhea incidence and mortality in children and adults, and the fifth most fatal pathogen among children, globally [3]. Notably, our study reflected the decline in rotavirus cases following the introduction of rotavirus vaccination in Germany in 2013, with rotavirus being detected in only one symptomatic child in our cohort. In parallel with the decline in rotavirus infections, norovirus emerged as the primary cause of acute pediatric gastroenteritis in our cohort, consistent with trends observed in other countries [14, 24]. Likewise, asymptomatic norovirus infections have been reported in previous studies from Ecuador and the Netherlands [27, 32].

Sapovirus and astrovirus were among the other viral pathogens identified as clinically relevant contributors to AGE within our cohort. We found comparable rates among symptomatic samples to those reported in recent American and Dutch studies [24, 27], and at low frequency among asymptomatic samples, which is consistent with findings from a Finnish publication [26]. Adenovirus was one of the most frequently found pathogens in our cohort of children with AGE, but it was also commonly detected in samples of healthy controls and had a weaker, non-significant association with symptoms. Findings indicating that adenovirus often causes asymptomatic infections align with earlier research [33, 34]. Additionally, adenovirus loads in AGE and healthy samples were similar in our cohort. Of note, our detection method targeted adenovirus F, which comprises two main types, enteric adenovirus serotypes 40 and 41, and not the broader spectrum of pan-adenovirus types. Nevertheless, given that various adenovirus serotypes have been shown to contribute differentially to AGE, a separate detection of unique serotypes would aid in the interpretation of these data [25].

This study indicates a minor contribution of bacterial pathogens to the development of AGE in children without significant comorbidities from high-income countries. Bacteria were widespread among all samples analyzed in our study (94 detections in 79/178 samples), yet the comparison of bacterial detection rates between asymptomatic and symptomatic samples revealed comparable frequencies, and therefore a low association with symptoms (OR 1.1, 95% CI 0.6–2.0). Comparison of bacterial loads via Ct values between symptomatic and asymptomatic samples did not show significant differences either. A possible explanation for these observations is the challenge of unambiguously defining pathogenic strains by targeting a single virulence gene. This is common in many clinical assays, including ours. The genomic heterogeneity of some bacteria requires the detection of multiple virulence genes to improve diagnostic precision, though consensus is lacking on which markers are most predictive of pathogenicity. The method employed in our study for detection of some bacteria may have led to the inclusion of non-pathogenic strains, which could have diluted the strength of the association with disease. These considerations are particularly relevant for the *E. coli* strains, which account for 59 of the 94 bacterial detections in this cohort.

Enteropathogenic *E. coli* (EPEC), the most prevalent bacterial pathogen detected, exhibited higher detection rates in healthy control samples compared to AGE cases. EPEC is part of a group of diarrheagenic *E. coli* strains that encompasses enteroaggregative *E. coli* (EAEC), enterotoxigenic *E. coli* (ETEC), and Shiga toxin-producing *E. coli* (STEC) [35]. Notably, in our study, STEC was absent and ETEC was found in only one asymptomatic sample. Conversely, EAEC was detected in symptomatic samples, but at a low frequency and consistently in conjunction with other pathogens, complicating its direct attribution to AGE. The presence of these bacteria in asymptomatic samples may be influenced by the molecular test used to detect these pathotypes. The diagnostic kit employed in this study targets the *eaeA* locus to detect EPEC, which is present in both typical and atypical strains, encompassing varying levels of pathogenicity. This broad detection complicates the interpretation of EPEC’s clinical significance [36, 37]. EAEC was identified via the detection of the *aggR* locus alone, which does not encompass all pathogenic EAEC strains and cannot, by itself, confirm the pathogenicity of the detected strain [20, 21, 38, 39]. Different considerations apply to *C. difficile*, another prevalent finding and an increasingly recognized emerging infectious agent causing AGE [40], especially in adult patients with prior antibiotic therapy. Detection of C. difficile toxin B, as used in our study, represents the established marker of pathogenicity and is endorsed in international guidelines [41, 42]. *C. difficile* was the second most widespread bacterial finding in our cohort, but also showed high rates of codetections and asymptomatic carriage, challenging its direct association with AGE. This may be related to the observation that asymptomatic *C. difficile* is relatively common in infants and toddlers [43], who represent the primary patient population studied here. Finally, parasitic detections were rare, with *Dientamoeba fragilis* being the only parasite observed, showing only modest, non-significant association with symptoms, a finding corroborated by the lack of symptom improvement following treatment in a randomized controlled trial [44].

Pathogen loads have been proposed to correlate with the clinical manifestation of AGE [45]. However, in our cohort, the majority of enteric pathogens exhibited substantial similarity in pathogen loads between symptomatic and asymptomatic samples. Notably, astrovirus, norovirus GII, and sapovirus were exceptions, supporting their stronger association with AGE.

The unique design of our study, which includes paired asymptomatic and symptomatic samples from the same individuals, allows the intra-individual comparison of pathogen dynamics over time. This approach provides valuable insights into the changes in pathogen presence before AGE and during AGE. Of note, while in 72% of children with AGE new pathogens were identified, a substantial proportion (28%) had no newly acquired pathogens, suggesting that factors beyond pathogen acquisition may contribute to disease onset. For example, differences in serotypes among microorganisms [46] or virulence acquisition, for example via horizontal gene transfer upon bacteriophage infection in bacteria [47], may contribute to the development of pathogenic traits in otherwise innocuous enteric microorganisms, leading to their ability to induce AGE. In addition, the presence of recurrent detections in nearly one-third of cases emphasizes the potential role of persistent or reactivated pathogens in AGE. Interestingly, 44% of children had pathogens in their asymptomatic sample that were no longer detectable during the illness, raising questions about transient colonization or competing pathogen dynamics. These findings, while derived from a high-income setting, support trends reported from both low- and high-income countries on the high rate of asymptomatic infections and codetections in children with gastroenteritis [28, 48], and underscore the importance of distinguishing between colonization and pathogenicity when interpreting results. Despite frequent identification of some enteropathogens in asymptomatic individuals, further research is needed to elucidate their clinical relevance and public health implications. One possible explanation for this phenomenon is the presence of immunity acquired from previous encounters with pathogens [33]. This study offers valuable data to inform future investigations into the global epidemiology of AGE. It highlights the complexity of pathogen-host interactions and emphasizes the need for longitudinal studies to unravel the mechanisms underlying symptomatic and asymptomatic infections. Continued investigation into pathogen-disease associations and factors influencing AGE development is essential for informing preventive and therapeutic strategies effectively.

While our study provides informative insights, limitations should be acknowledged. First, the relatively small sample size limits the robustness and broader applicability of our findings. Second, our cohort was restricted to children without significant comorbidities, introducing potential selection bias and restricting the generalizability of our findings to children with underlying health conditions. Third, although our diagnostic method covered a broad range of common AGE pathogens, it may have missed other potential pathogens, such as enterovirus [19]. Additionally, while the detection method used has high sensitivity, it cannot differentiate between strains or serotypes with varying pathogenicity. Lastly, the study was geographically focused, which limits the transferability of results of detection rates of specific pathogens to regions or settings with different epidemiological profiles. For example, the low detection of rotavirus in our cohort reflects countries with rotavirus vaccination programs, distinguishing them from other high-income countries without such preventive measures.

## Conclusion

In conclusion, our study provides insights into the etiology of pediatric acute gastroenteritis in young children in high-income countries by examining the pathogen detection rate in asymptomatic individuals and comparing it with symptomatic samples from the same children. While viral pathogens, with the exception of adenovirus F, showed strong associations with symptoms, bacterial and parasitic pathogens were common in asymptomatic individuals and not significantly associated with clinical symptoms, questioning their relevance in high-income countries. These insights have the potential to enhance our understanding of the role of various microorganisms in gastrointestinal disease, develop more targeted preventive and therapeutic interventions, reduce the harms of overdiagnosis and unnecessary treatment, and ultimately mitigate the burden of AGE in pediatric populations.

## Supplementary Information

Below is the link to the electronic supplementary material.

Supplementary Material 1

## Data Availability

Data generated in this study are included in this published article (and its supplementary information files). Raw data are available from the corresponding author upon reasonable request.
