# Supplementary material for: Detection of enteric pathogens in young children before and during acute gastroenteritis: results from a prospective German birth cohort study (LoewenKIDS)
Source: Infection. 2025 Oct 20;54(1):353–64. doi: 10.1007/s15010-025-02670-1 (PMC12864203; doi:10.1007/s15010-025-02670-1)

Supplementary Information

**Detection of enteric pathogens in young children before and during acute gastroenteritis: results from a prospective German birth cohort study (LoewenKIDS)**

Chiara Lincetto ^a^#, Felipe Romero-Saavedra ^a^, Diana Laverde ^a^, Riccardo Lincetto ^b^, Melanie Meyer-Buehn ^a^, Bianca Klee ^c^, Cornelia Gottschick ^c^, Rafael Mikolajczyk ^c^, Johannes Huebner ^a^, Tilmann Schober ^a^

^a^ Division of Pediatric Infectious Diseases, Dr. von Hauner Children's Hospital, Ludwig Maximilians University, Munich, Germany

^b^ Independent Researcher

^c^ Institute for Medical Epidemiology, Biometrics and Informatics (IMEBI), Interdisciplinary Centre for Health Sciences, Medical Faculty of the Martin Luther University Halle-Wittenberg, Halle (Saale), Germany

#Address correspondence to Chiara Lincetto, chiara.lincetto@med.uni-muenchen.de.

**Supplementary Fig. 1** Seasonality of AGE within the Loewenkids sub-cohort. **a** Number of children presenting with AGE per month. Data is presented as the distribution of the collected symptomatic samples by month. **b** Monthly detection rate of individual enteric pathogens. For each pathogen, symptomatic (dashed orange bars) and asymptomatic (dotted green bars) detection rates are stacked by month. Detection rate is calculated as the percentage of samples positive for each pathogen among all pathogen-positive samples in that month. Vertical dashed lines denote seasonal transitions (spring, summer, autumn, winter)

a


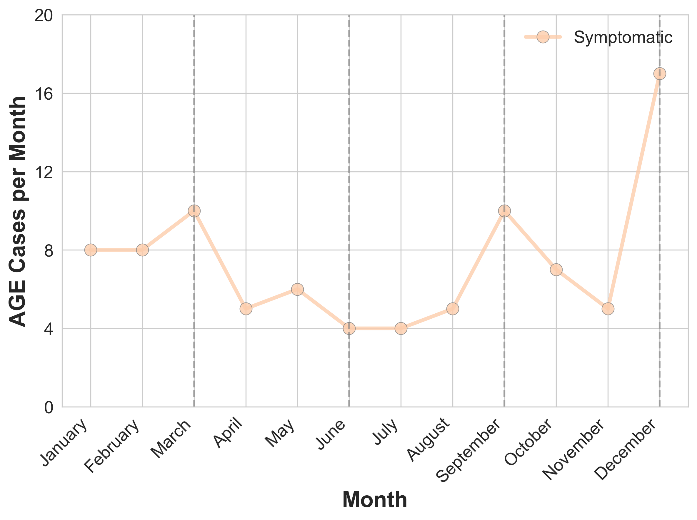


b


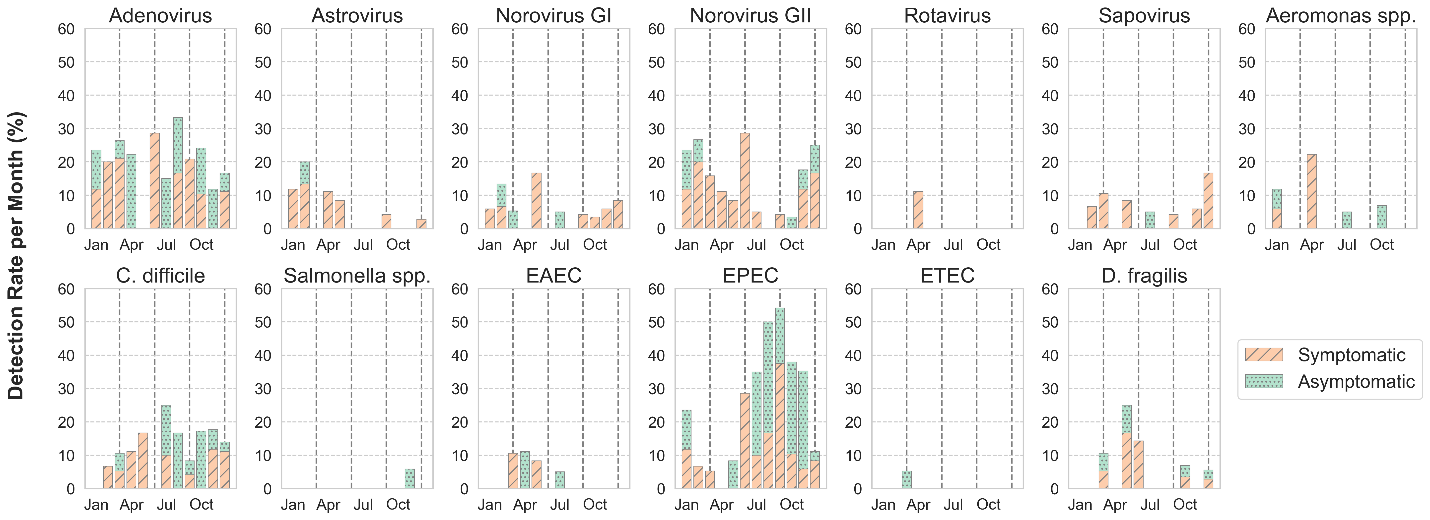


**Supplementary Fig. 2** Scatter plot of Principal Component 1 (PC1) and PC2 based on Principal Component Analysis (PCA) of the pathogen detection profile of each sample, including codetections and quantitative pathogen load. PC1 explains 12% of the variance, while PC2 explains 11% of the variance in the data. Each point represents a sample, colored and shaped by clinical presentation (asymptomatic or symptomatic). Grey lines connect each sample to its corresponding K-means cluster centroid (grey X), representing the geometric center of each identified cluster


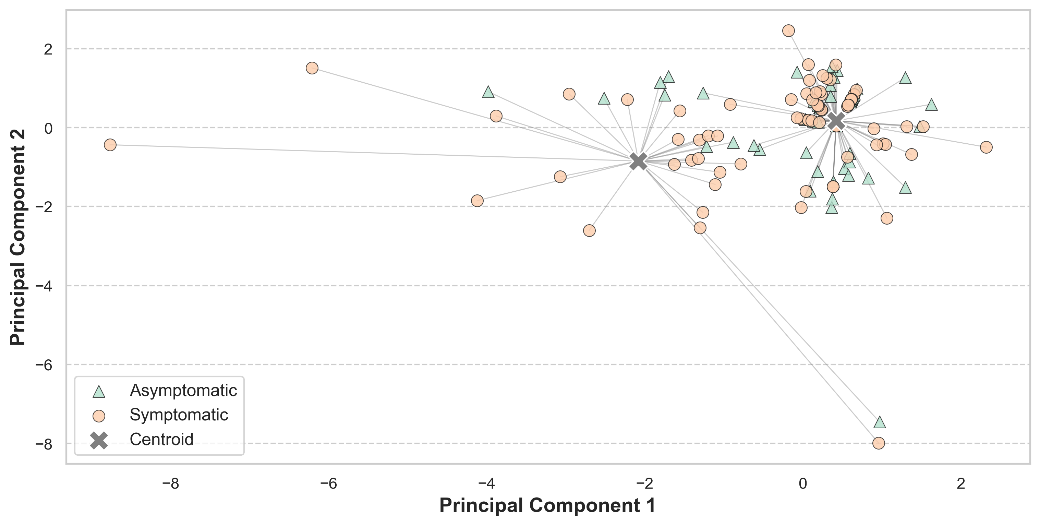

Supplement: Supplementary file 1 — Supplementary Material 1 [file 15010_2025_2670_MOESM1_ESM.docx]
